# Epigenetic Insights Into Necrotizing Enterocolitis: Unraveling Methylation-Regulated Biomarkers

**DOI:** 10.1007/s10753-024-02054-x

**Published:** 2024-05-30

**Authors:** Bowen Tian, Xiaogang Xu, Lin Li, Yan Tian, Yanqing Liu, Yide Mu, Jieting Lu, Kai Song, Junjian lv, Qiuming He, Wei Zhong, Huimin Xia, Chaoting Lan

**Affiliations:** 1 The First School of Clinical Medicine, Southern Medical University, Guangzhou, Guangdong China; 2 Department of Pediatric Surgery, Guangdong Provincial Key Laboratory of Research in Structural Birth Defect Disease, Guangzhou Women and Children’s Medical Center, Guangdong Provincial Clinical Research Center for Child Health, Guangzhou Medical University, No.9 Jinsui Road, Zhujiang New Town, Tianhe District, Guangzhou, Guangdong China; 3 Department of Anesthesiology, Jiangxi Provincial Children’s Hospital, Nanchang, Jiangxi China

**Keywords:** DNA methylation, single-cell transcriptomics, necrotizing enterocolitis, multiomics network, methylation-regulated genes

## Abstract

Necrotizing enterocolitis (NEC) is a multifactorial gastrointestinal disease with high morbidity and mortality among premature infants. This study aimed to identify novel methylation-regulated biomarkers in NEC intestinal tissue through multiomics analysis. We analyzed DNA methylation and transcriptome datasets from ileum and colon tissues of patients with NEC. We identify methylation-related differential genes (MrDEGs) based on the rule that the degree of methylation in the promoter region is inversely proportional to RNA transcription. These MrDEGs included *ADAP1, GUCA2A, BCL2L14, FUT3, MISP, USH1C, ITGA3, UNC93A* and *IL22RA1*. Single-cell data revealed that MrDEGs were mainly located in the intestinal epithelial part of intestinal tissue. These MrDEGs were verified through Target gene bisulfite sequencing and RT-qPCR. We successfully identified and verified the *ADAP1*, *GUCA2A*, *IL22RA1* and *MISP*, primarily expressed in intestinal epithelial villus cells through single-cell data. Through single-gene gene set enrichment analysis, we found that these genes participate mainly in the pathological process of T-cell differentiation and the suppression of intestinal inflammation in NEC. This study enhances our understanding of the pathogenesis of NEC and may promote the development of new precision medicine methods for NEC prediction and diagnosis.

**Supplementary Information:**

The online version contains supplementary material available at 10.1007/s10753-024-02054-x.

## INTRODUCTION

Necrotizing enterocolitis (NEC) is the most common life-threatening gastrointestinal emergency in neonatal intensive care units [1]. Symptoms of NEC, such as feeding intolerance, may begin slowly and insidiously, but they can quickly develop into catastrophic fulminant NEC [2]. The estimated overall mortality rate for diagnosed NEC is approximately 25%, increasing to 50% in extremely preterm infants requiring NEC surgery [3]. The survivors will experience a range of short- and long-term complications, including bowel dysfunction, severe sepsis, impaired growth, microcephaly, and significant neurodevelopmental delays [4, 5]. Multiple risk factors are involved in the development of NEC, including preterm birth, formula feeds, low oxygen levels, dysregulation of gut bacteria, and infections [1]. "Immature intestinal barrier development with a strong immune response to intestinal inflammation" is the core pathological reaction of NEC, but the mechanism of this pathological process is still unclear [6]. The clinical symptoms of NEC are nonspecific and difficult to distinguish from those of other diseases with similar characteristics, so early and accurate diagnosis is difficult. Therefore, searching for potential biological markers of NEC will contribute to the timely diagnosis of NEC and help improve the prognosis of newborns with NEC [7]. Given the complexity of NEC pathogenesis and disease progression, using multiomics and multilevel analyses, including epigenomics, transcriptomics, and single-cell mapping, can help us enhance our understanding of its pathophysiological mechanisms and explore new potential diagnostic biomarkers.

Epigenomics refers to changes in gene expression based on the absence of changes in the nucleotide sequence of a gene. There are currently three main forms: DNA methylation, histone modification, and noncoding RNA regulation. Changes in DNA methylation have been applied to diagnose some malignant tumors and inflammatory diseases, and have good diagnostic efficiency. Studies have revealed extensive hypomethylation in the blood and tumor tissues of patients with colorectal cancer. Methylation of the *VIM* and *SEPT9* genes had good diagnostic efficacy for colorectal cancer, while hypermethylation of the *SFRP2* gene is an early diagnostic feature of colorectal cancer [8–10]. In ulcerative colitis (UC), the intestinal epithelium is highly methylated, and the highly methylated *ESR-1* and *N-33* genes can be used as diagnostic markers for UC [11–14]. At present, multiomics analysis, a method for comprehensively understanding biological systems through the comprehensive use of multilayer biological data, is also widely used in studying disease mechanisms, diagnosis and other fields. The combination of epigenetics and transcriptomics revealed that the *S100A9* gene in the gut of inflammatory bowel disease (IBD) patients is characterized by high DNA methylation and low RNA expression, and was identified as a new diagnostic marker [15]. After comprehensive analysis of GWAS data, DNA methylation data and transcriptome data, a gene regulatory network was constructed, and *PSMB9* and *CD74* were mined and identified as new biomarkers for type two diabetes mellitus [16]. Good M’s group reported hypermethylation in the ileum and colon tissues of patients with NEC lesions, and hypermethylation of intestinal epithelial cells was a characteristic manifestation of NEC [17, 18]. In this study, we combined DNA methylation and transcriptomic analysis methods to explore hypermethylated regulatory genes in the intestinal tract of NEC lesions. We verified their potential as new biomarkers of NEC using clinical samples. Furthermore, after labeling the distribution of target genes in intestinal tissues by single-cell mapping, single-gene GSEA was used to analyze the possible mechanism pathways involved.

**Fig. 1:**
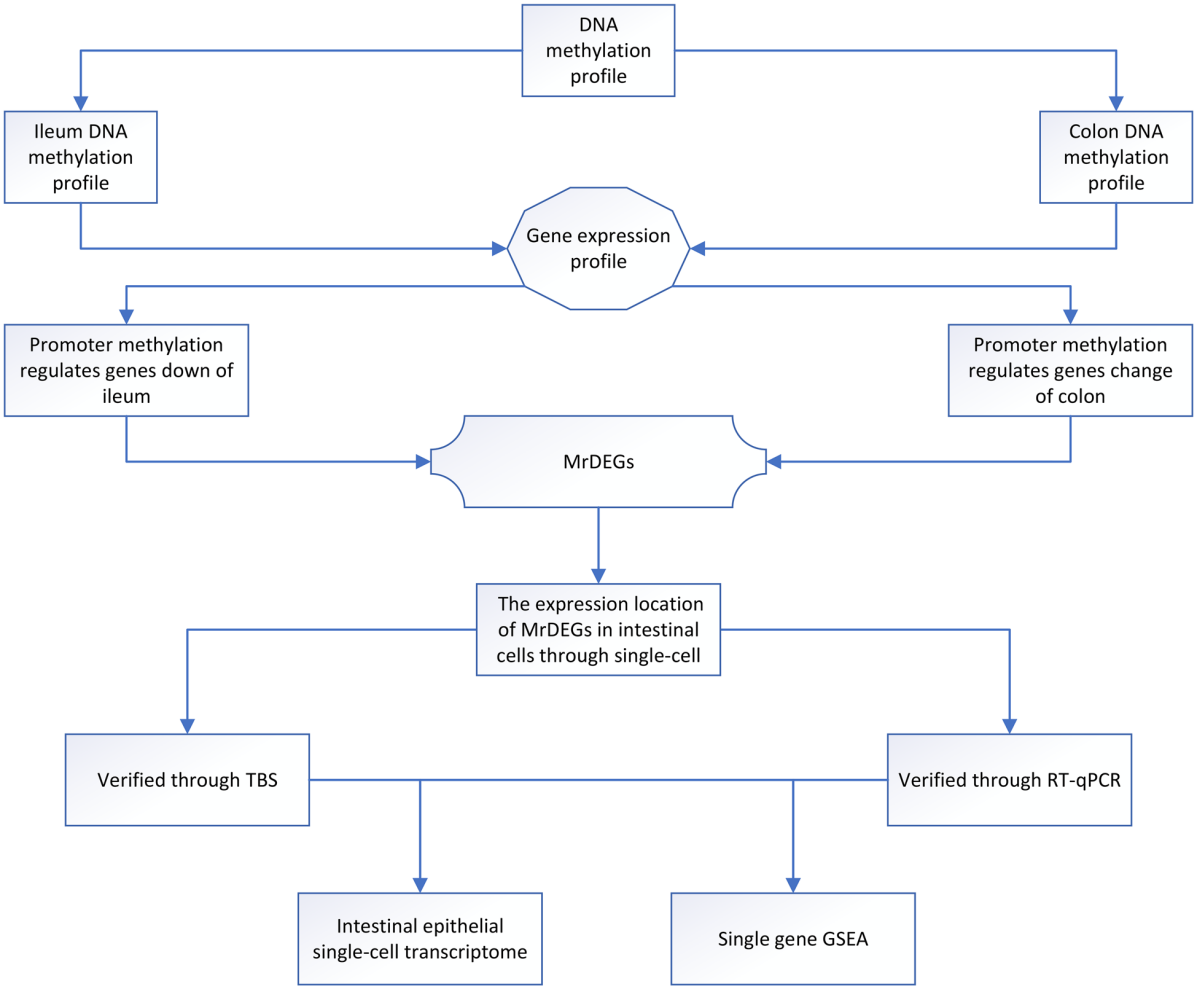
The outline of the analysis pipeline. The analysis pipeline of the study is shown graphically to increase organization and readability. MrDEGs: methylation-related differential genes.

Here, we identified hypermethylation features in the ileum and colon of patients with NEC lesions, and through multiomics analysis, we found that the *ADAP1*, *GUCA2A*, *IL22RA1* and *MISP* genes can serve as diagnostic biomarkers for NEC. Moreover, we investigated the expression of MrDEGs mainly in the villous epithelial cells of intestinal tissue through single-cell transcriptomics. Additionally, we explored whether MrDEGs are associated with the core pathogenesis of immature intestinal barrier development and a strong immune response to intestinal inflammation in patients with NEC, and the results were validated through GSEA (Fig. 1).

## MATERIALS AND METHODS

### Ethical Approval

The Helsinki Declaration and the ethical standards of the Guangzhou Women and Children’s Medical Center Institutional Review Committee (No. 2016042036) were followed during the collection of human samples for this study. We collected a total of 16 intestinal tissue samples. This included eight ileal tissue samples from infants affected by NEC. In addition, we selected eight normal ileal tissue samples from postoperative NEC patients with intestinal fistulas as controls, ensuring that these samples were matched based on gestational age.

### Data Sources

The gene expression and DNA methylation profiles of NEC were collected from the Gene Expression Omnibus (GEO) database. Inclusion criteria were: (1) Describe the sample organization's originating location. (2) Three suitable datasets were chosen: GSE212913 (ileum) [18], GSE212997 (colon) [19], and GSE46619 (colon & ileum) [20]. Single-cell data were obtained from Adi Egozi’s group [21]. Single cell sequencing was performed twice for each intestinal tissue region in the single cell dataset, once from CD45^+^ and CD45^−^ cells within CD45^−^ cells isolated from intestinal tissue using the MACS cell separation system enriched with CD45^−^ microspheres, and once from the entire single cell suspension of the tissue digest without any selection. Table 1 presents all of the data's specifics.

**Table 1:** Basic Information of the Selected Datasets

**Dataset**	**Platform**	**collection method**	**Tissue (Homo sapiens)**	**Samples (number)**			**Experiment type**	**Author**
				**Total**	**NEC**	**Non-NEC**		
**GSE212997**	GPL16791	surgery	colon	21	8	13	Methylation profiling by high throughput sequencing	Good M
**GSE212913**	GPL16791	surgery	ileum	15	6	9	Methylation profiling by high throughput sequencing	Good M
**GSE46619**	GPL6244	surgery	intestine	9	5	4	Expression profiling by array	NG PC
https://doi.org/10.5281/zenodo.5813397		surgery	small intestine	11	6	5	Single Cell RNA by Seurat	Adi Egozi

### DNA Methylation Data Processing

The raw DNA methylation data (GSE212913 and GSE212997) were downloaded in COV file format. To determine DNA methylation signatures, we employed the beta-binomial test using the R packages DSS (2.48.0). Differentially methylated regions (DMRs) were identified with DSS [22], by comparing biological replicates in a two-group setup, and the ‘DMLtest’ and ‘callDMR’ functions were utilized. Visual representation of DMRs methylation levels was achieved using the R package ggplot2. Additionally, we analyzed DMRs for genomic annotation by querying UCSC (https://genome.ucsc.edu/cgi-bin/hgTables) to retrieve hg38.knownGene information. The promoter region was defined as the 2 kb upstream of the transcription start site. Genomic annotation priority followed this order: promoter, coding exon, 5’ UTR, 3’ UTR, intron, and intergenic regions, all visualized using ggplot2.

### Microarray Data Processing of Differentially Expressed Genes (DEGs)

The raw data (GSE46619) was retrieved as a MINIML file. It comprises of data recorded by the platform, samples, and GSE. Normalize the collected data using log2 transformation. Based on the platform file annotation information, use the limma (3.56.2) program in R software to select DEGs with adjusted *p* < 0.05 and absolute multiple change > 1 [23]. R program created a heatmap and a volcano graphic.

### Methylation-Related Genes and UpSet Diagrams

Differentially methylated genes regulated by methylation were determined based on the methylation sites, methylation differences, and RNA expression differences. The results were visualized using the UpSet (1.4.0) package in R software [24].

### Immune Infiltration Analysis

To analyze immune infiltration, we utilized data from GSE46619 and uploaded it to ImmuCellAI (http://bioinfo.life.hust.edu.cn/web/ImmuCellAI). This platform allowed us to estimate the infiltration abundance of 36 types of immune cells based on the gene expression profile derived from the microarray data. Following normalization of the gene expression matrix, we conducted immune infiltration analysis via ImmuCellAI. Intergroup comparisons were conducted by the Wilcoxon rank-sum test. Furthermore, we employed Spearman correlation analysis to investigate the potential associations between NEC and various immune cells.

### Single-Cell Analysis

Single-cell data were analyzed using the Seurat package (3.2.2) in R software. All subjects were merged, and cells with fewer than 1900 UMIs, fewer than 1000 genes per cell, or a mitochondrial fraction greater than 30% were filtered out. Cells with a fraction of erythrocyte markers above 10% were also excluded. The remaining cells were normalized and scaled using the SCTransform function, with regression of the sum of UMIs. Principal component analysis (PCA) was performed based on variable genes, excluding mitochondrial and ribosomal genes. According to an article by the Adi Egozi group, the name of each cluster was assigned based on the top 10 markers of each cluster [21]. The process was repeated for normalization, scaling, and PCA calculations.

### Gene Set Enrichment Analysis (GSEA) and Single Gene GSEA

A cohort of 5 patients from the GSE46619 dataset was analyzed, and the patients were divided into two groups based on the median expression value of one hub gene. Specifically, we selected annotated gene sets from c2.cp.kegg.v2023.1.Hs.entrez.gmt in the Molecular Signatures Database (MSigDB) using the GSEA package (1.64.0) in R software [25]. The permutation type employed was phenotype. Our GSEA results were filtered using the following criteria: normalized enrichment score (NES) > 1.0, false discovery rate (FDR) *q* > 0.25, and nominal *p* < 0.05. The hallmark gene sets showing significant enrichment were examined, and gene set enrichment plots were generated.

### Visualizing DNA Methylation Sites

The UCSC website (https://genome.ucsc.edu/) was used to visualize highly methylated regions. The displayed features include the following: (1) GENCODE Genes: This track combines high-quality manual annotations with evidence-based automatic annotations of the entire human genome produced by the GENCODE project. (2) RefSeq: RefSeq provides a matched collection of high-confidence transcripts that are annotated similarly in both RefSeq (NCBI) and Ensembl/GENCODE (headed by EMBL-EBI). (3) ENCODE cCREs: This track contains a subset of typical DNase hypersensitive sites from ENCODE and Roadmap Epigenomics samples, as supported by histone modification (H3K4me3 and H3K27ac) or CTCF-binding data. (4) DMR: We analyzed different methylation regions.

#### Hematoxylin–Eosin Staining

The ileum tissue from neonatal necrotizing enterocolitis lesions and matched samples of the same gastric age from necrotic NEC patients were soaked in 10% neutral buffered formalin for 24 h. Afterward, the samples were dehydrated using a graded alcohol dilution solution. Subsequently, the tissue was embedded in wax and sliced. Finally, the paraffin sections were stained with H&E, and pathological changes in the two groups of ileal tissues were observed using an optical microscope (Leica DM4B, Germany).

#### Targeted Bisulfite Sequencing (TBS)

DNA was extracted from cells or tissue samples using an Omega DNA Extraction kit. E-Gene Co., Ltd. in Shenzhen, China performed target gene bisulfite sequencing. Primers were designed to avoid amplification bias between methylated and unmethylated sequences. For BSP validation, genomic DNA (500 ng) was obtained using the ZYMO EZ DNA Methylation Gold Kit™ (Zymo Research, California, USA). PCR amplification was carried out, and the products were used for library construction and sequenced on an Illumina HiSeq platform.

#### RT-qPCR

According to the manufacturer’s instructions, total RNA was extracted using Trizol reagent (Biosharp, Hefei, China), and the RNA ratio of 260/280 (NanoDrop ND1000, Wilmington, DE, USA) was confirmed to be qualified within the range of 1.8 and 2.0. Subsequently, 1000 ng of RNA was reverse transcribed into cDNA using the Quantscript RT assay kit. RT-qPCR was performed using the SYBR Green assay kit and ABI Prism 7500 sequence detection equipment (Applied Biosystems, Foster City, CA, USA). All PCR data were normalized to gene expression levels of glyceraldehyde 3-phosphate dehydrogenase (GAPDH). The 2-ΔΔ Ct technology was used to determine the relative expression of genes related to the reference gene, and this process was repeated three times [26]. The primer sequence was shown in Table 2.

**Table 2:** Basic Information on the RT-qPCR Analyses

**GENE**		**Primer sequence (5'─3')**
ADAP1	F	TTGAGCCGGAAGTTTGTGCT
	R	GCTCGGAGTGCATTGAACCA
GUCA2A	F	GGGTTGGGAAACTCAGGAACT
	R	AGTTCGGGTTGCTACAGAGG
FUT3	F	AGCTGTCCTCATCCACTGCTC
	R	TGGGGGAAACAGGTGATTGGT
IL22RA1	F	CGCACCTACCAAATGCACCT
	R	GGTGCCAAGGAACTCTGTGT
MISP	F	TGCCCATCGAGGAGTCAGAG
	R	AGACCTCTGGGCTTACTGCC
USH1C	F	TCCATCAAAGTGAGACACATCG
	R	CCGACACAAACTGATCCACATA
ITGA3	F	GCGCAAGGAGTGGGACTTAT
	R	TGAAGCTGCCTACCTGCATC
UNC93A	F	TTCTTCGCCAGCTGGTACAC
	R	TGTTTCCCGTGATCGTGAGG
GADPH	F	TCTGACTTCAACAGCGACAC
	R	CGTTGTCATACCAGGAAATGAG

#### Immunofluorescence Staining

Paraffin Sects. (4 mm) of ileal tissue were prepared and fixed with 4% PFA. The cells were blocked with 5% goat serum in a constant temperature and humidity incubator at 37 ℃ for 1 h, and the diluted primary antibody (Immunoway, State of Texas, USA) was kept in a dark room at 4 ℃ overnight. After washing with TBST and incubation with secondary antibodies (ThermoFisher, Massachusetts, USA) for 1 h at room temperature, the cells were washed again, incubated in the dark with DAPI blocking solution for 10 min, and anti-fluorescence attenuating sealant (Solarbio, Beijing, CN) was added. Immunofluorescence images were obtained using a Leica sp8 inverted fluorescence microscope. The mean fluorescence intensity of IL22RA1 was calculated using ImageJ software.

#### Statistical Analysis and Code

The data is shown as mean ± standard error (SEM). We used Student's t-test (for two groups) or one-way analysis of variance (ANOVA) with Bonferroni post hoc correction. All analyses were carried out using R (version 4.2.3) or GraphPad Prism 5 (GraphPad Software, San Diego, CA, USA). All the code used for analysis in R language is included in the supplementary file 2.

## Results

### Characteristics of DNA Methylation in Colonic and Ileal NEC Lesions

**Fig. 2:**
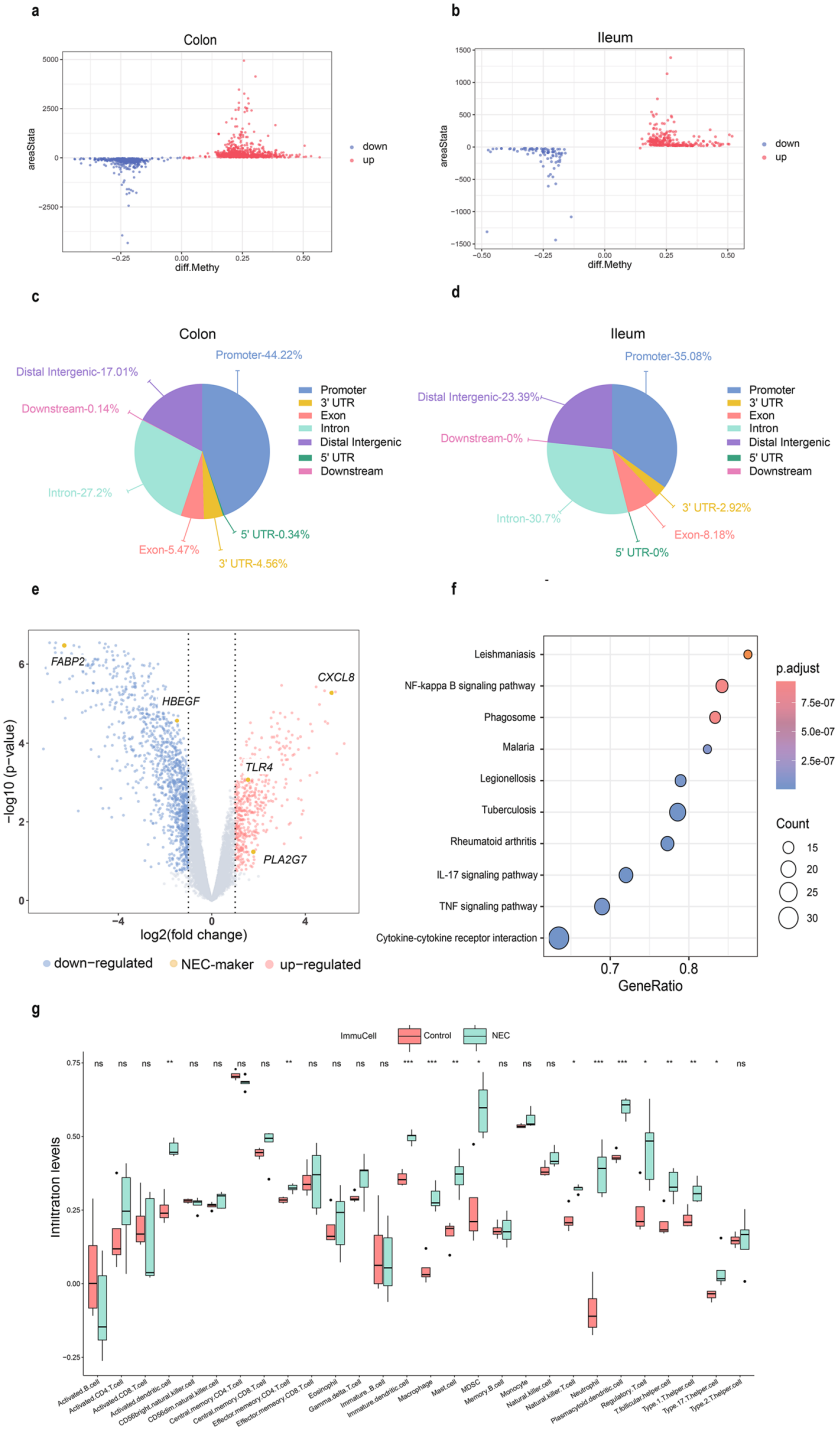
Differentially methylated regions in human NEC from the GSE212997 and GSE212913 datasets and differentially expressed genes in human NEC from the GSE46619 dataset. **a-b** Scatter plot of DMRS in human NEC and CTRL samples. The abscissa is the average methylation level in the two conditions, and the ordinate represents the difference in methylation level between the two conditions. The red dots represent the significantly upregulated regions, and the blue dots indicate the significantly downregulated regions. **c-d** The sector plots represent the proportion of DMRs in different regions: promoter, exon, intron, intergenic, 3’-UTR and distal intergenic regions. **e** Volcano plot of the DEGs associated with NEC marker genes. **f** Gene Set Enrichment Analysis of DEGs. **g** A box plot displaying the proportion of immune cells predicted by immune infiltration analysis between NEC and control groups. The vertical axis displays the enrichment level of immune cells, and the horizontal axis displays the name of each type of immune cell. The top number of each group of cells indicates whether there is a significant difference in the enrichment of this type of immune cell between the two groups using t-test validation. Red represents the control group, and green represents the NEC group, ***p < 0.001, **p < 0.01, *p < 0.05, ns: no significant difference. NEC: Necrotizing enterocolitis, CTRL: Control, DMR: Differentially methylated regions, DEG: differentially expressed genes.

We analyzed the DNA methylation data of the colon and ileum separately using the DSS package, and annotated the data using the UCUS website. To obtain information on DMRs, we visualized the average methylation level (diff. Methy) under two conditions and the difference in methylation levels between colonic and ileal tissues (areaStat) through scatter plots (Fig. 2a-b). Overall, we found that the NEC tissues in both the ileum and colon were hypermethylated, which is consistent with the findings of Good M’s research group [17]. In the ileum, the methylation level of 357 regions changed, of which the methylation level decreased in 95 regions and increased in 262 regions. There were more regions in the colon where the methylation level changed, with a total of 1550 regions, of which the methylation level decreased in 556 regions and increased in 994 regions. Since methylation has different effects on the expression of downstream genes at different parts of the gene, it is currently widely recognized that an increase in the methylation ratio of the promoter will silence the expression of downstream genes. Therefore, we visualized the distribution of DMRs in the genes and found that the proportion of methylation in the promoter region was the highest in both intestinal segments. The proportion of methylation regions in the promoter region in the colon was 45% (702/1550), and the proportion of methylation regions in the promoter region in the ileum was 34.35% (123/357) (Fig. 2c-d).

### Immunological Characteristics of NEC Transcriptome Data

At the same time, to study the impact of DNA methylation on downstream RNA, we analyzed the GSE46619 transcriptome dataset, which includes five NEC samples, with four human ileum samples and one human colon sample. We screened the common DEGs between the colon and ileum. The upregulated and downregulated DEGs were visualized through volcano plots. The analysis revealed 1693 DEGs, including 499 upregulated genes and 1194 downregulated genes, which are known marker genes for NEC, namely, *TLR4, CXC-8, PLA2/G7, HBEGF* and *FABP2* [27–31] (Fig. 2e). To determine the biological functions of the DEGs, we used GSEA to analyze the pathways associated with the DEGs between the NEC disease group and the healthy control group, and visualized the top 10 pathways in descending order of p-value with dot plots. The gene sets related to immune inflammation are the NF-κB signaling pathway, IL-17 signaling pathway, and TNF signaling pathway (Fig. 2f). Since immune-related factors play important roles in the pathogenesis of NEC, some overactivated immune cells can have a destructive effect on the intestine. By analyzing the differences in the distribution of immune cells between the NEC group and the control group through immune infiltration analysis of this dataset, we found that the numbers of activated dendritic cells, effector memory CD4^+^ T cells, immature dendritic cells, macrophages, mast cells, MDSCs, natural killer T cells, neutrophils, plasmacytoid dendritic cells, regulatory T cells, T follicular helper cells, Th1 cells and Th17 cells in the NEC group were different from those in the control group (Fig. 2g).

### Joint Multi-Omics Analysis of MrDEGs in NEC

**Fig. 3:**
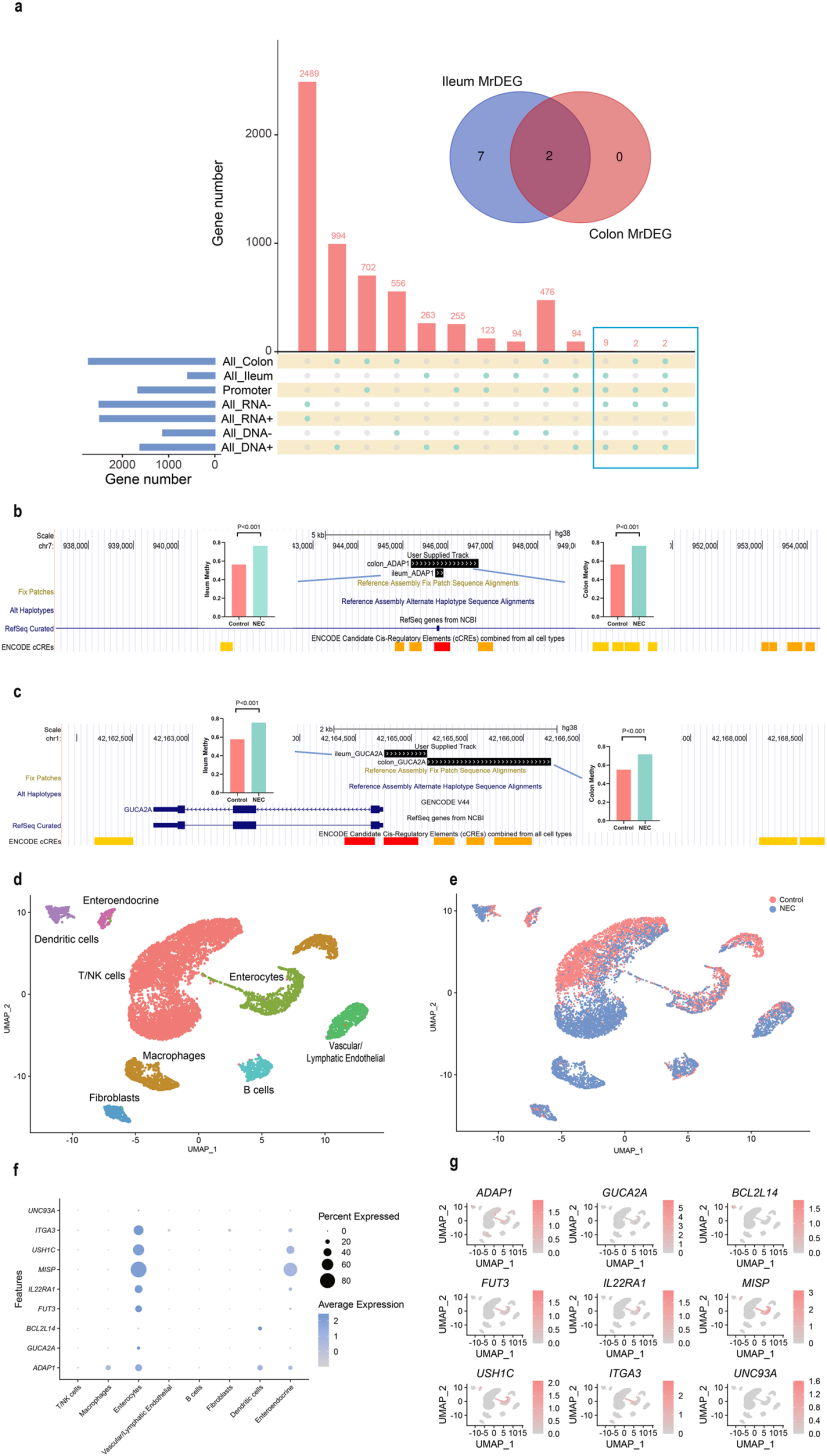
Screening for MrDEGs and exploring MrDEG locations through NEC single-cell datasets. **a** The UpSet map shows the genes that conform to the upregulation or downregulation of methylation-related genes at different intestinal sites. **b-c** The methylation region of ADAP1/GUCA2A was mapped onto the genome (using custom tracks in the UCSC genome browser). The black area represents the location of high methylation, and the bar graph shows the average methylation level of the NEC group compared to the control group. **d** Single-cell atlas classified by cell type. **e** Single cell diagram classified by disease. **f** Dot plot showing the classification of MrDEGs by cell type. **g** UMAPs colored by MrDEGs. MrDEGs: methylation-related differentially genes.

To understand whether differentially expressed genes are regulated by DNA methylation during the onset of NEC, we combined DNA methylation and transcriptomics analysis results to integrate the results of the two different analyses to determine the key genes regulated by methylation. High transcription can be attributed to a decrease in DNA methylation at the promoter, resulting from epigenetic modification [32]. Therefore, we screened genes related to methylation regulation based on whether the DMR was located on the promoter and whether the gene expression level was negatively correlated with DNA methylation. There were 9 genes in the ileum, namely *ADAP1*, *GUCA2A*, *BCL2L14*, *FUT3*, *MISP*, *USH1C*, *ITGA3*, *IL22RA1* and *UNC93A*. In the colon, there were two genes *GUCA2A* and *ADAP1*, both expressed in the ileum and colon (Fig. 3a). The methylation sites of these two genes are not consistent in the colon or ileum. We visualized the methylation sites through the UCSC website (Fig. 3b-c, Fig. S1). At the same time, the NEC single-cell transcriptomics data helped us understand the expression of MrDEGs in different intestinal cells. First, after processing, we separated eight different cell types from the NEC intestinal tissue single-cell data shared by Adi Egozi’s group, including macrophages, dendritic cells (DCs), B cells, T/natural killer (NK) cells, vascular/lymphatic endothelial cells, fibroblasts, intestinal secretory cells and other intestinal cell populations (Fig. 3d). We then calculated the relative numbers of these cell types in the NEC group and the control group (Fig. 3e). We analyzed the expression abundance of nine MrDEGs in each cell population, except that *BCL2L14* was expressed mainly on dendritic cells, while the remaining genes were expressed mainly on intestinal epithelial cells and some on intestinal secretory cells (Fig. 3f-g).

### Verification of MrDEG DNA Methylation and Transcriptome Levels

**Fig. 4:**
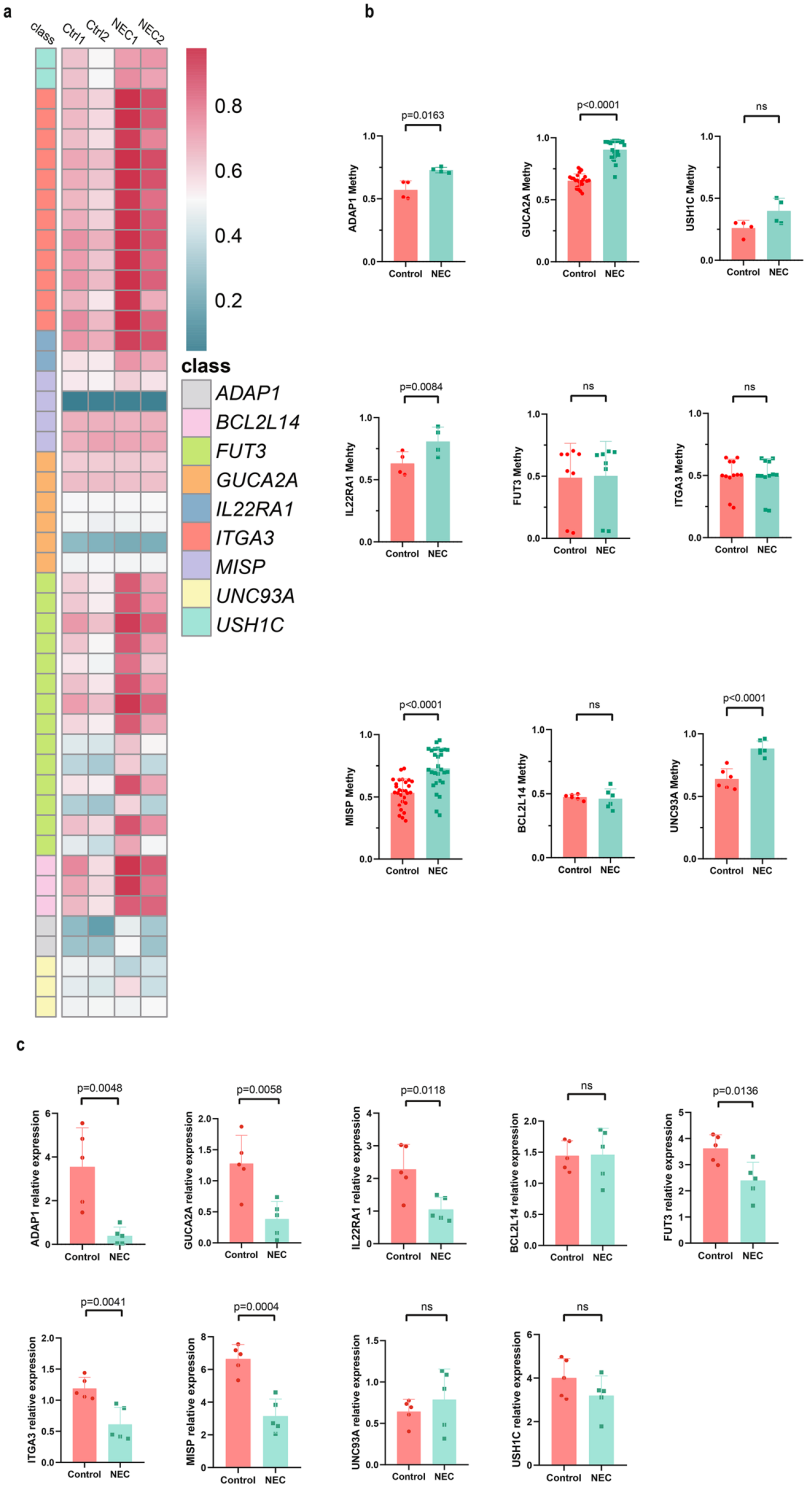
The methylation level and RNA expression of MrDEGs were verified in human tissue specimens. **a** Visualization of the methylation levels of MrDEGs. The heatmap shows the methylation level of CpG islands, and the right side indicates the starting position of sequencing of this part of the CpG island. **b** Histograms showing the differences in the methylation levels of MrDEGs. **c**The mRNA expression levels of methylation-related genes in NEC patients. MrDEGs: methylation-related differentially genes.

To substantiate the results of our previous analysis, we conducted a TBG experiment on the first 2 kb of nine MrDEGs using ileal tissue samples from NEC-affected infants at 28 and 34 weeks of gestation, and matched samples from the same gestational age from postoperative NEC patients with intestinal fistulas as controls. The results indicated that *ADAP1*, *GUCA2A*, *IL22RA1*, *MISP*, *UNC93A* and *USH1C* had increased methylation levels in NEC (Fig. 4a-b). Interestingly, the length of the methylation regions of different genes detected via targeted bisulfite sequencing (TBS) was similar to the length of the methylation regions of each gene according to our bioinformatics analysis. For instance, *ADAP1* had a shorter methylation region, while *GUCA2A* had a longer methylation region. This consistency further validated our findings and underscored the potential importance of these genes in the context of NEC. In our subsequent step, we performed an RT-qPCR experiment to compare the RNA expression in 5 ileal tissue samples from NEC-affected infants at 30–32 weeks of gestation with matched samples from the same gestational age from postoperative NEC patients with intestinal fistulas as controls. The RT-qPCR results showed a decrease in the expression of *ADAP1*, *GUCA2A*, *IL22RA1*, *FUT3*, *ITGA3* and *MISP* in NEC (Fig. 4c). After validation at the methylation and RNA levels, *ADAP1*, *GUCA2A*, *MISP* and *IL22RA1* were MrDEGs found to be that were inversely proportional to DNA methylation and transcription levels.

### MrDEG Function Prediction

**Fig. 5:**
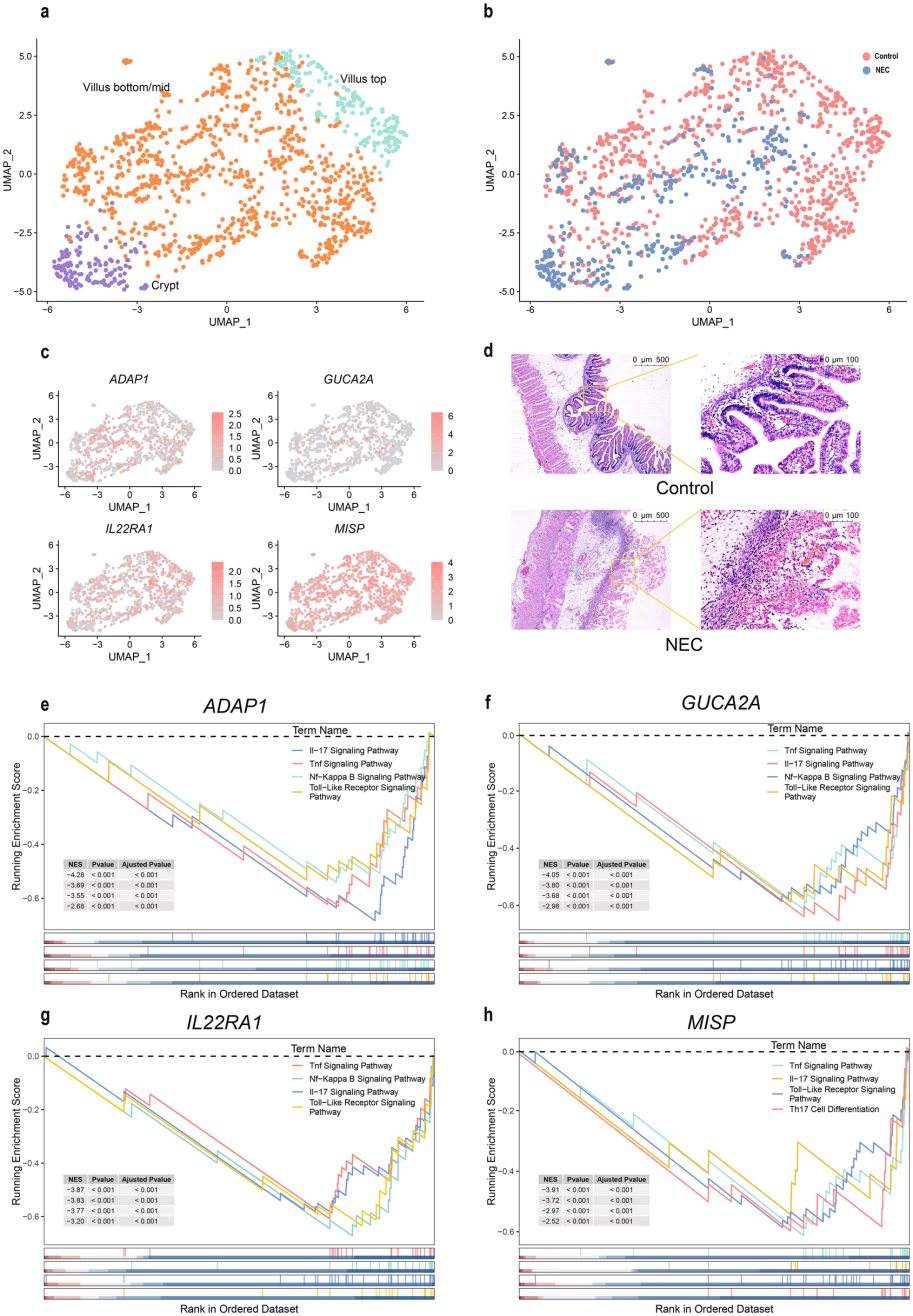
Predicting the localization and role of MrDEGs through single-cell data and single-gene GSEA. **a** Reclustered atlas of the enterocyte cluster classified by cell type. **b** Reclustered atlas of the enterocyte cluster classified by disease. **c** UMAPs colored by MrDEGs. **d** Observing the pathological changes in the villous structure of NEC and the control group through H&E staining, the left image shows a 5 × view, and the right image shows a 20 × view. **e-h** Immune-related gene sets in the group of MrDEGs. MrDEGs: methylation-related differentially genes.

To understand the role of MrDEGs in the pathological process of NEC, it is necessary to explore the specific expression locations of these four genes in intestinal tissues. According to the previous single-cell results, these four genes are mainly concentrated in the intestinal epithelial cell cluster. Therefore, the intestinal epithelial cell cluster was reclustered, and the cell distribution of the NEC and control groups was displayed. The cells were then separated into three subclusters based on the markers of each subcluster: crypt cells, villus top cells, and villus bottom/mid cells (Fig. 5a**-**b). The results of MrDEGs in these three cell categories showed that *ADAP1*was more highly expressed in villus bottom/mid cells and crypt cells, but less expression is highly expressed in the crypt cells. The overall distribution of *GUCA2A* was lower and concentrated on the villus bottom/mid cell. *IL22RA1* and *MISP* were similar, and both were concentrated on the villus cells (Fig. 5c). Immunofluorescence staining was used to demonstrate the difference and location of IL22RA1 gene in NEC and control intestinal segment tissue (Fig. S2). We used the ileum of 28-week-old NEC patients and matched samples of the same gestational age from postoperative NEC patients with intestinal fistula as controls for HE staining. The results showed that the villous structure of NEC tissue was severely damaged (Fig. 5d). To understand the functional changes caused by MrDEGs, we used single-gene GSEA, divided GSE46619 into a high expression group and a low expression group according to four MrDEGs. We identified the top three gene sets related to immunity in ascending order of P value. As the expression of *ADAP1* increased, the expression of genes involved in the TNF signaling pathway, NF-kappa B signaling pathway, IL-17 signaling pathway and Toll-like receptor signaling pathway decreased (Fig. 5e). Similarly, when *GUCA2A* and *IL22RA1* were highly expressed, the TNF signaling pathway, NF-kappa B signaling pathway, IL-17 signaling pathway, and the toll-like receptor signaling pathway decreased (Fig. 5f-g). When *MISP* expression increases, TNF signaling pathway activity, IL-17 signaling pathway activity, Toll-like receptor signaling pathway activity and Th17 cell differentiation decreased (Fig. 5h). Notably, under conditions of NEC, the expression of MrDEGs decreased, and the enriched inflammatory pathway was overactivated, causing intestinal cell damage.

## Discussion

NEC presents a clinical challenge due to its high incidence and mortality rate. Furthermore, the lack of highly sensitive and specific biomarkers, coupled with nonspecific clinical symptoms observed in children, complicates early diagnosis of NEC. Through DNA methylation and transcriptomic multiomics analysis, we identified nine genes with elevated DNA methylation and decreased RNA transcript levels: *ADAP1*, *GUCA2A*, *BCL2L14*, *FUT3*, *MISP*, *USH1C*, *ITGA3*, *IL22RA1* and *UNC93A*. Single-cell mapping revealed that in addition to *BCL2L14*, which is primarily expressed in dendritic cells, the remaining genes are predominantly expressed in intestinal epithelial sites. After double verification with TBS and RT-qPCR, we confirmed that four genes are regulated by DNA methylation: *ADAP1, GUCA2A, IL22RA1* and *MISP*. Current research suggests that the pathogenesis of NEC is mainly due to overactivation of immune inflammation centered on TLR4, dysbiosis of the intestinal microbiota, and severe damage to the intestinal villus structure caused by mesenteric vasculature ischemia, leading to irreversible intestinal barrier damage [33]. We used GSEA to explore the links in which MrDEGs may participate during the onset of NEC.

By analyzing DNA methylation data from patients with NEC, we verified that NEC is generally hypermethylated in both colon and ileum tissues, consistent with the conclusion of the GOOD M’s study [17]. The microbial abundance in colon tissue is much greater than that in ileum tissue, and an increase in intestinal microbial abundance promotes DNA methylation [34]. Our results confirmed more DMRs in colon tissue than in ileum tissue. The relationship between DNA methylation and the expression of downstream genes in diseases is currently one of the hotspots of research. A large amount of evidence shows that as the density of CpG dinucleotides in the promoter increases, the degree of gene silencing increases, and the inhibitory effect of promoter methylation on transcription plays an important role in various diseases, such as gastric cancer and thyroid cancer [35, 36]. We found that the proportion of methylation in the promoter region was the highest in both the colon and ileum tissues of patients with NEC, so it is necessary to explore the genes regulated by methylation in NEC tissues. Overactivation of immune inflammation is one of the core mechanisms of NEC onset. We analyzed the immunological characteristics of NEC through transcriptomics. The high expression of differentially expressed genes, such as *TLR4, CXC-8, PLA2/G7, HBEGF* and *FABP2*, and the overactivation of immune pathways shown by GSEA prove that immune disorders are involved in the onset of NEC. Moreover, through immune infiltration analysis, we found that the numbers of dendritic cells, neutrophils, MDSCs, and T cells in the NEC group significantly increased.

*ADAP1* (ArfGAP with Dual PH domains 1) is protein-coding gene primarily expressed in the brain and colon. It is the GTPase activating protein (GAP) of the small GTPase ARF6. Initially, considered a neuron-restrictive factor, *ADAP1* was later found to amplify specific T-cell signaling programs, playing a crucial role in the progression of HIV disease [37]. A genetic association study involving nearly 30,000 IBD patients identified *ADAP1* as a new biomarker for inflammatory diseases [38]. Th17 cells play a central role in regulating intestinal homeostasis and pathogen elimination, while Treg cells suppress autoimmunity and are responsible for tolerance to self-antigens [39]. Disruption of the Th17/Treg balance can lead to the development of many inflammatory diseases, including NEC. IL2, a key immune regulatory factor, can reduce Th17 and promote the activity of Treg cells [40]. In NEC research, it has been proposed that a reduction in Treg cells is strongly correlated with IL2 levels [41]. Kuropka B’s group showed that a lack of *ADAP1* reduces T-cell activation by lowering IL2 [42]. We hypothesize that the downregulation of *ADAP1* expression due to increased methylation levels, leading to a decrease in IL2 transcription, results in a decrease in the proportion of Treg cells, which may be involved in the pathogenesis of NEC.

*GUCA2A* (guanylate cyclase activator 2A) is a ligand for guanylate cyclase-C, and is primarily highly expressed in the small intestine and colon. It is one of the main participants in the transmembrane receptor guanylate cyclase-C (GC-C) signaling pathway. GC-C maintains the intestinal functional barrier by regulating tight junction proteins and mucous secretion by goblet cells [43, 44]. The number of tight junction proteins and goblet cells is significantly reduced in NEC lesion intestines, causing impairment of the intestinal functional barrier [45]. Our analysis suggested that a decrease in *GUCA2A* expression due to high DNA methylation may lead to a decrease in GC-C levels. *GUCA2A* is associated with intestinal health, intestinal permeability, and inflammation, and is used for disease monitoring and treatment guidance, especially for intestinal diseases such as rectal cancer and IBD [46]. According to an immunohistochemical analysis of NEC intestinal tissue, GUCA2A had a marginal effect (*p* = 0.053) in the NEC group (n = 16) compared to the control group (n = 11) [47]. Our multiomics analysis confirmed a significant difference in *GUCA2A* between intestinal tissue from patients with NEC lesions and control intestinal tissue, suggesting that *GUCA2A* is a potential biomarker for NEC.

*IL22RA1* (interleukin 22 receptor subunit alpha 1) belongs to the class II cytokine receptor family. It is a specific receptor for interleukin 22 (IL22), which is only expressed in nonimmune cells, such as epithelial cells, endothelial cells, and fibroblasts. IL22 originates from Th1 cells, Th17 cells, and innate lymphoid cells (ILCs), and mainly plays an anti-inflammatory role in the intestine [48]. Thus, IL22-IL22RA1 is a specific signaling mediator between inflammatory cells and nonimmune cells in NEC. After IL22 binds to *IL22RA1* located in Paneth cells, it can regulate Th17-related immune responses and microbial group colonization to promote the recovery of intestinal immune function [49]. IL22-IL22RA1 can promote the remission of colitis in patients with ulcerative colitis by increasing the expression of mucus-related molecules and restoring the production of mucus by goblet cells [50]. Supplementing NEC model mice with IL22 can effectively alleviate colitis [51]. We speculate that the methylation-regulated IL22RA1 signaling pathway may participate in the pathogenesis of NEC by regulating Th17 immune-related responses and mucus barrier function.

*MISP* (mitotic spindle positioning) is a myosin bundling protein that is mainly expressed in the apical membrane of the intestine, is involved in determining cell morphology and mitosis, is necessary for the correct positioning of the mitotic spindle, and plays an important role in the formation and stability of microvilli [52]. Currently, research on *MISP* in intestinal inflammatory diseases is relatively rare. In a mouse model induced by sulfated polysaccharide, *MISP* plays a role in the recovery of the colon after inflammation through its anti-inflammatory and proliferative effects, and a lack of *MISP* exacerbates colitis in mice, which may be a new therapeutic target for treating IBD [53]. We speculate that the increase in *MISP* methylation leads to a decrease in intestinal gene expression, leading to the destruction of the top villus structure in NEC and a decrease in intestinal anti-inflammatory and proliferative functions, thus contributing to NEC inflammation.

The occurrence and development of NEC are closely related to intestinal epithelial cells. One of the pathological features of NEC is severe destruction of the intestinal epithelial villus structure. Through single-cell data, we found that these four MrDEGs were expressed mainly in the villus cells of intestinal epithelial cells. When the methylation level is high in intestinal tissue, the expression of four genes is downregulated, leading to destruction of the intestinal barrier, overactivation of immunity, and aggravation of intestinal inflammation. Based on single-gene GSEA, we found that the low expression of MrDEGs was mainly related to the following immune pathways. First, because of the overactivation of the TLR4-TNF-NFκB signaling pathway, many studies have shown that TLR4 is one of the core factors of NEC, and after inhibiting the expression of TLR4-TNF-NFκB, intestinal inflammation in NEC patients is relieved [28]. In addition, the immune pathway closely related to the pathogenesis of NEC involves the activation of Th17 cell differentiation and the Th17 secretory factor IL17 signaling pathway, which is consistent with the increase in the proportion of Th17 cells in the immune infiltration analysis results described above. We have previously described that the lack of *ADAP1* and *IL22RA1* in other intestinal inflammatory diseases has been confirmed to promote Th17 differentiation. Our GSEA results are consistent with these findings and suggest that *GUCA2A* and *MISP* may potentially affect Th17 cell differentiation.

Formula feeding is one of the risk factors for NEC [6]. It has been reported that the global DNA profile of the mouse liver is demethylated during breast-feeding, whereas following weaning, DNA methylation rises. The addition of PPAR-α ligands to milk powder reduced DNA methylation in formula-fed mice [54]. The ingredients of commercial formula milk are constantly being optimized, such as increasing the content of lactoferrin and α-linolenic acid to meet the enteral nutritional needs of preterm infants. Nutritional ingredients that can reduce DNA methylation in preterm infants may be added in the future formula update, which will help to reduce the hypermethylation of intestinal tissue and reduce the risk of NEC. Currently, there are no market-oriented drugs targeting *IL22RA1*, *ADAP1*, *MISP* and *GUCA2A*. This study provides clues for future drug development of NEC.

**Fig. 6:**
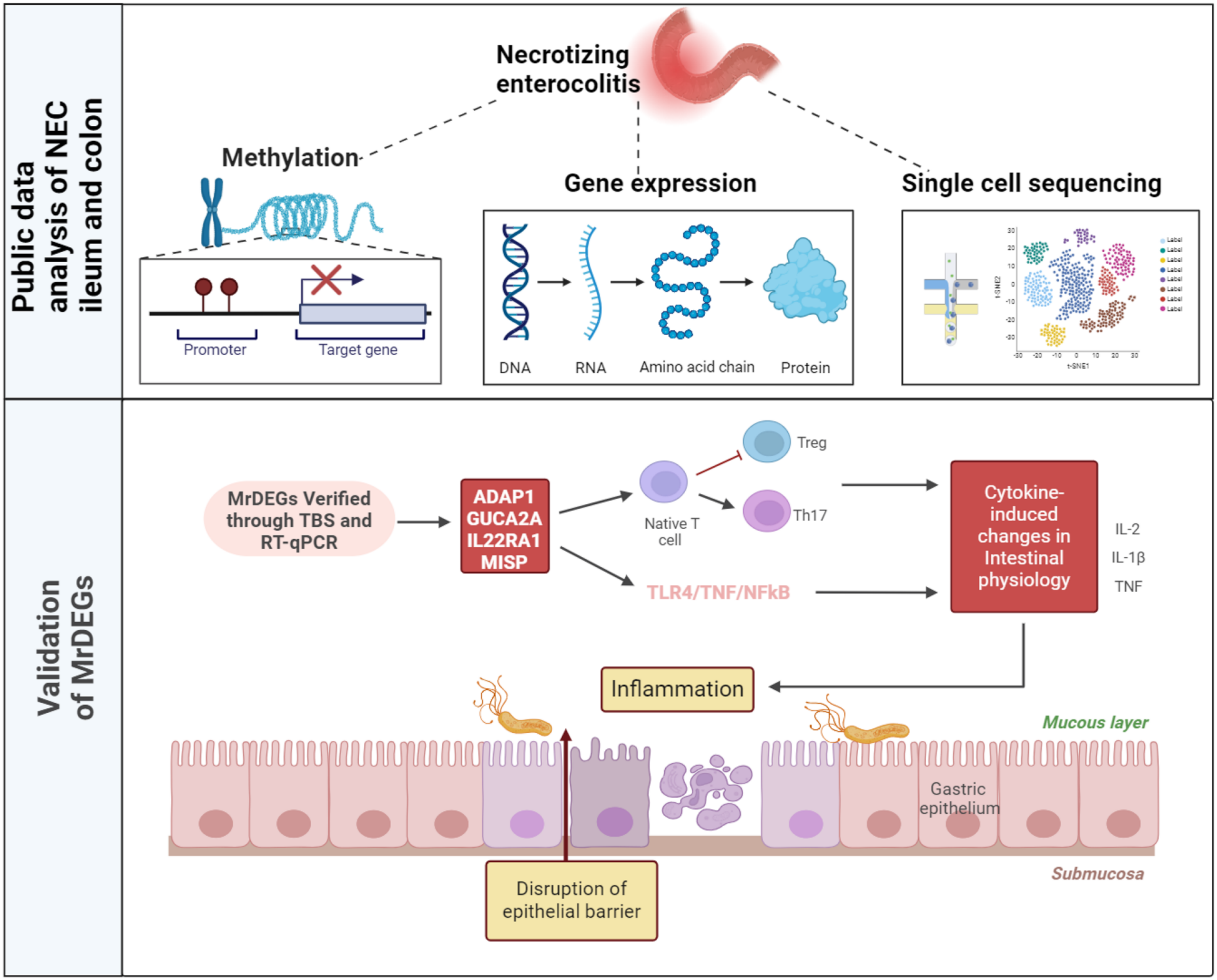
Schematic diagram of the screening process and mechanism of action of MrDEGs.

There are several shortcomings in this study. First, we need to experimentally verify our findings in a larger clinical cohort of patients with NEC in the future. Second, we need to continue to explore the specific role of MrDEGs in the pathogenesis of NEC in the future (Fig. 6).

## CONCLUSION

In summary, through multiomics analysis of DNA methylation, transcriptome, and single-cell atlas data, this study identified novel NEC-related genes potentially regulated by DNA methylation, namely, *ADAP1*, *GUCA2A*, *IL22RA1* and *MISP*, and their aberrant promoter CpG methylation status in NEC was further verified. The involvement of these genes in the pathogenesis of NEC, including the overactivation of inflammatory pathways and the promotion of Th17 cell differentiation and proinflammatory cytokine secretion, was further speculated by bioinformatical approaches. This will allow us to further understand the pathogenesis of NEC and help discover new diagnostic markers for NEC in clinical practice.

## Supplementary Information

Below is the link to the electronic supplementary material.

Supplementary file1 (TXT 25 KB)

Supplementary file2 (PDF 346 KB)

## Data Availability

The data are available from the authors upon reasonable request.
