# Supplementary material for: Epigenetic Insights Into Necrotizing Enterocolitis: Unraveling Methylation-Regulated Biomarkers
Source: Inflammation. 2024 May 30;48(1):236–53. doi: 10.1007/s10753-024-02054-x (PMC11807086; doi:10.1007/s10753-024-02054-x)
Supplement: Supplementary file 2 — Supplementary file2 (PDF 346 KB) [file 10753_2024_2054_MOESM2_ESM.pdf]

Epigenetic Insights into Necrotizing Enterocolitis: Unraveling Methylation-Regulated Biomarkers

Bowen Tian[1]*, Xiaogang Xu[1]*, Lin Li[2]*, Yan Tian[3], Yanqing Liu[2], Yide Mu[2], Jieting Lu[1], Kai Song[2], Junjian lv[2], Qiuming He[2], Wei Zhong[2#], Huimin Xia[1,2#], Chaoting Lan[2#]

1. The First School of Clinical Medicine, Southern Medical University, Guangzhou, Guangdong, China

2. Department of Pediatric Surgery, Guangdong Provincial Key Laboratory of Research in Structural Birth Defect Disease, Guangzhou Women and Children's Medical Center, Guangzhou Medical University, Guangdong Provincial Clinical Research Center for Child Health, No.9 Jinsui Road, Zhujiang New Town, Tianhe District, Guangzhou, Guangdong, China.

3. Department of Anesthesiology, Jiangxi Provincial Children's Hospital, Nanchang, Jiangxi, China.

*These authors contributed equally.

#Corresponding authors:

Wei Zhong, Guangzhou Women and Children's Medical Center, Guangzhou Medica University, No.9 Jinsui Road, Zhujiang New Town, Tianhe District, Guangzhou, Guangdong, China, zhongwei@gwcmc.org

Huimin Xia, Guangzhou Women and Children's Medical Center, Guangzhou Medical University, No.9 Jinsui Road, Zhujiang New Town, Tianhe District, Guangzhou, Guangdong, China, xia-huimin@foxmail.com

Chaoting Lan, Guangzhou Women and Children's Medical Center, Guangzhou Medical University, No.9 Jinsui Road, Zhujiang New Town, Tianhe District, Guangzhou, Guangdong, China, lanctlyq@foxmail.com, 0086-13826079458

Fig. S1: Visualization of differentially methylated regions: (a-g) The methylated regions of BCL2L14, FUT3, MISP, USH1C, ITGA3, IL22RA1, and UNC93A are mapped onto the genome using custom orbitals in the UCSC Genome Browser. The black area represents the location of high methylation, and the bar graph shows the average methylation level of the NEC group compared to the control group.

Fig. S2: To demonstrate the specific expression location of IL22RA1 in intestinal tissue. IL22RA1 was stained by immunofluorescence in ileum of NEC and control group, the bar graph presents the IL22RA1 fluorescence intensity of NEC versus control under three identical areas in two sets of slides, and the differences were compared using t-test.blue is DAPI, green represents IL22RA1, Scale bar, 50μm.

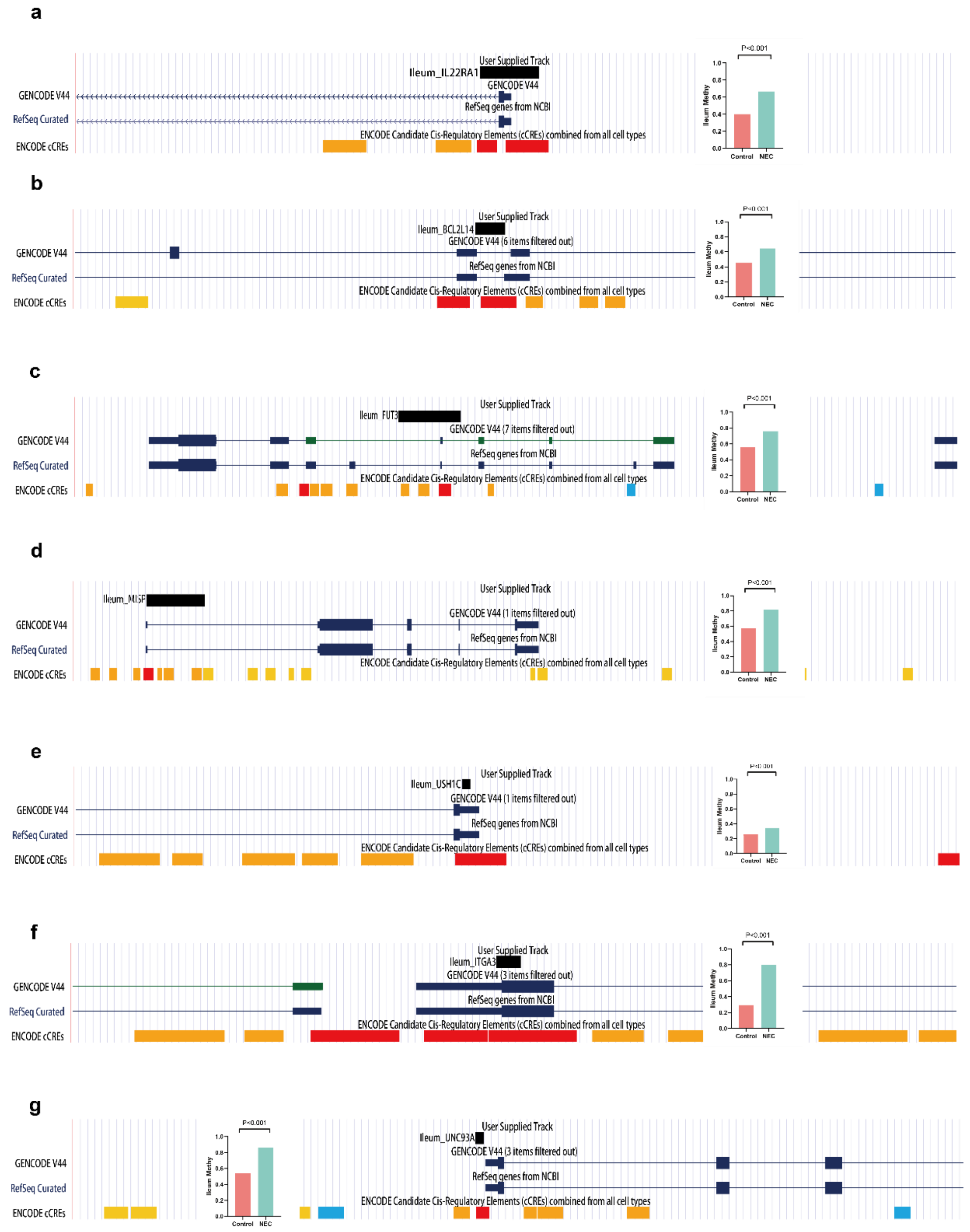

Fig. S1: Visualization of differentially methylated regions.

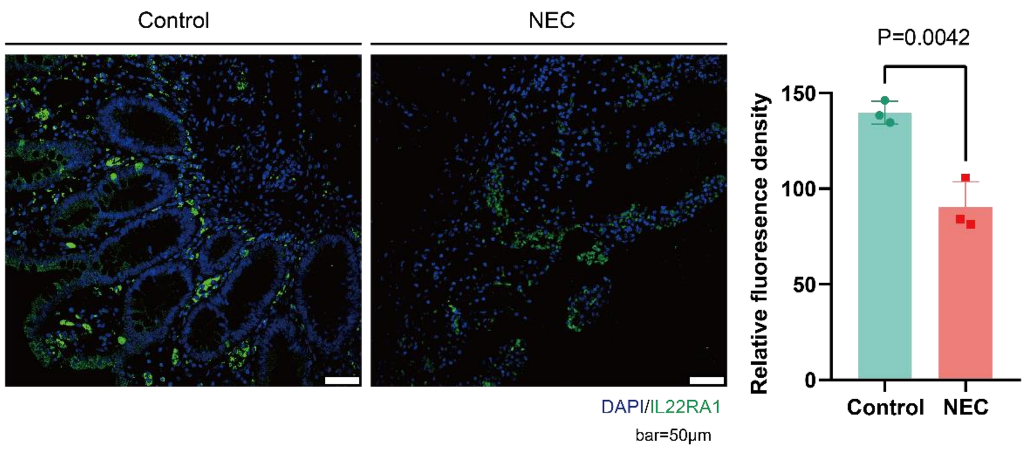

Fig. S2: To demonstrate the specific expression location of IL22RA1 in intestinal tissue.
